# A Casparian strip domain-like gene, *CASPL*, negatively alters growth and cold tolerance

**DOI:** 10.1038/srep14299

**Published:** 2015-09-24

**Authors:** Jinghua Yang, Changqing Ding, Baochen Xu, Cuiting Chen, Reena Narsai, Jim Whelan, Zhongyuan Hu, Mingfang Zhang

**Affiliations:** 1 Laboratory of Germplasm Innovation and Molecular Breeding, Institute of Vegetable Science, Zhejiang University, Hangzhou, 310058, P. R. China; 2 Key laboratory of Horticultural Plant Growth, Development & Quality Improvement, Ministry of Agriculture, Hangzhou, 310058, P. R. China.; 3 Department of Animal, Plant and Soil Science, School of Life Science, Australian Research Council Centre of Excellence in Plant Energy Biology, LaTrobe University, Bundoora, Victoria 3086, Australian

## Abstract

A cold-induced transcript encoding a Casparian strip membrane domain (CASP)-like protein (*ClCASPL*) was identified in watermelon (*Citrullus lanatus)*. Fluorescence microscopy analysis showed that ClCASPL-GFP is localized in the plasma membrane. The orthologous gene in *Arabidopsis thaliana* (*AtCASPL4C1*) was also found to play an important role in cold tolerance. Expression analysis using a *β*-glucuronidase (GUS) reporter reveals that *AtCASPL4C1* is widely expressed in a variety of organs and is cold inducible. Analysis of *AtCASPL4C1* T-DNA knock-out plants showed altered growth dynamics, faster growth, increased biomass (dry weight) and earlier flowering compared to wild type (Col-0) and *ClCASPL* overexpressing plants. *AtCASPL4C1* knock-out plants showed elevated tolerance to cold stress, while overexpressing *CICASPL* resulted in increased sensitivity to cold stress in *Arabidopsis*. Interestingly, *AtCASPL4C1* knock-out plants did not display significant alterations in the Casparian strip formation in roots. Thus, the combination of these results suggests a role for *CICASPL* and *AtCASPL4C1* beyond Casparian strip formation in roots, possibly indicating a more fundamental role in vascular tissue.

Abiotic stresses are major environmental factors that adversely affect plant growth, development and yield. Plants have evolved complex signaling networks to adapt to abiotic stresses via modulating various physiological and biochemical processes^1^,^2^,^3^. These stress signals are perceived by receptors, transduced and propagated by downstream effectors, ultimately altering the expression of a variety of genes that determine growth, tolerance and/or survival depending on the severity of the environmental conditions^2^,^4^.

Transmembrane (TM) proteins located in the plasma membrane are known to have diverse physiological functions including signal perception and recognition, via ion and metabolite exchange. In *Arabidopsis*, approximately 6,500 proteins are predicted to be TM proteins^5^. Some integral membrane proteins are induced by stress, such as drought stress in *Triticum turgidum*^6^, or salt stress in *Xerophyta viscose*^7^ as well as having a role during development, e.g. kernel development in *Hordeum vulgare*^8^.

The Casparian strip, first described by Robert Caspary in 1865^9^, is a ring-like cell wall structure in the root endodermis of vascular plants. The role of the Casparian strip is to block the passive flow of materials in vascular plants^10^. Recent reports showed that the Casparian strip is composed of a lignin polymer without suberin in *Arabidopsis*^11^. This lignin polymer structure generates a para-cellular barrier, analogous to tight junctions in animals, that is thought to be crucial for selective nutrient uptake, exclusion of pathogens, and many other processes^10^. Casparian strip membrane domain proteins (CASP, CASP1/2/3/4/5) are crucial for Casparian strip formation in the endodermis in plants. CASP1/2/3/4/5 belong to ‘uncharacterized protein family’ UPF0497 (39 members) in *Arabidopsis*^9^. These CASPs are proposed to form an extensive, transmembrane polymeric platform and were speculated to guide the assembly and activity of lignin biosynthetic enzymes^9^. Recently, evolutionary analysis of CASP family genes indicated that CASPL genes belong to the MARVEL (MAL and related proteins for vesicle trafficking and membrane link) protein family, which has only been experimentally described in metazoans, to date^12^.

Previously, we identified a gene encoding a cold-induced integral membrane protein in watermelon^13^. The orthologue to this gene was identified to be *At3g55390* in *Arabidopsis thaliana*, where it is annotated as the CASP-LIKE PROTEIN 4C1. *At3g55390* belongs to the CASP protein family containing the five CASP genes (*CASP1/2/3/4/5)*, which are known to mediate Casparian strip formation in plants^9^. Thereafter, these are referred to as *ClCASPL* (from watermelon) and *AtCASPL4C1* (CASP-like) or *AtCASPL4C1* from Arabidopsis, respectively. In this study, we investigated the role of *ClCASPL* and *AtCASPL4C1* in growth and cold tolerance in watermelon and Arabidopsis, respectively.

## Materials and Methods

### Plant Materials and treatment

An IVSM-9 inbred line of watermelon (*Citrullus lanatus)* and wild type (WT) of Arabidopsis (*Arabidopsis thaliana)* (Col-0) were used for amplification of *ClCASPL* and *AtCASPL4C1* (*At3G55390*) genes. WT *Arabidopsis thaliana* (Col-0) was used for transformation to construct *OX-ClCASPL* of Arabidopsis. The SALK_034800C line was used for screening of homozygous *Atcaspl4c1* knock-out mutant plants. 3-week-old plants in Jiffy seedling culture substrate or 2-week-old seedlings cultured in 1/2 MS medium of WT, *Atcaspl4c1* and *OX-ClCASPL Arabidopsis* were used for analysis of cold stress and phenotypes evaluations. Tobacco (*Nicotianaben thamiana*) was used for transient expression of the *ClCASPL* gene.

5 days old plants were transferred onto half-strength MS (Murashige-Skoog, sigma-Aldrich) medium and grew under 10 °C, light/dark (16 h/8 h) conditions. For soil growth plants, 21 days old plants were used to cold treatments, under 10 °C, light/dark (16 h/8 h) conditions. The pictures and data were collected at the indicated time. The values are means ± SD (n = 20). Bar = 1 cm. Star signs indicate a significantly difference (p < 0.05, student’s *t* test).

### Phylogenetic Tree Construction

The ClustalW program was used for alignment of *ClCASPL* with the *AtCASPL4C1* protein sequence, which was obtained from the TAIR database. After alignment by ClustalW, a Neighbour-Joining tree was constructed by using MEGA 6.0, with 1000 as the number of bootstrap replications. The 39 members of CASP family genes were included from TAIR database. The TMHMM program was used for transmembrane region identification for *ClCASPL* and *AtCASPL4C1*.

### Transient Expression of *ClCASPL* Gene in Tobacco

The amplification of *ClCASPL* coding sequence without a termination codon was linked to pMDC83 binary expression vector to generate the *ClCASPL*-pMDC83 by Gateway cloning^14^. The ClCASPL-GFP plasmid was introduced into *Agrobacterium tumefaciens* strain (GV1301). Leaves from tobacco (*Nicotianaben thamiana*) were used for transformation and for checking transient expression according to the procedures^15^. Various organelle-targeted markers fused with RFP were used as controls to determine sub-cellular localization^16^. These tobacco leaves were observed using a confocal laser scanning microscope (ZEISS). The primers used in this study for GFP fusion are listed in Supplementary Table 1.

### Expression of *CASPL*

*In silico* transcript abundance analysis for genes encoding proteins of the CASP family was carried out as outlined in Narsai *et al*.^17^. The promoter sequences were searched by A Database of Plant Promoter Sequences (http://linux1.softberry.com/berry.phtml?topic=plantprom&group=data&subgroup=plantprom). The promoter region of *At3g55390* was amplified and inserted into a region upstream of the GUS gene of within the pMDC162 binary expression vector using the gateway system^14^. The resulting construct was transformed in the *Agrobacterium tumefaciens* strain GV3101. Transformation of Arabidopsis was conducted according to the floral-dip method^18^. Tissues from transgenic plants were collected in microcentrifuge tubes. Subsequently, the samples were stained as previously published procedures^19^.

Cold-induced expression of *CASPL* was analyzed using qPCR and *CASPL*-promoter analysis using *ClCASPL* promoter-GUS activity after cold stress. GUS activity was measured as previously published procedures^20^. The primers used for promoter amplification are listed in Supplementary Table 1.

### AtCASPL4C1 and OX-ClCASPL Construction

cDNA amplification of the *ClCASPL* gene was inserted to pMDC32 binary expression vector to generate the *ClCASPL*-pMDC32 by gateway approaches^14^. The *ClCASPL*-pMDC32 plasmid was introduced into *Agrobacterium tumefaciens* strain (GV1301). The floral dipping method was used to generate *OX-ClCASPL* of *Arabidopsis*^18^. For screening of transgenic plants, seeds harvested from transformed *Arabidopsis* plants were surface-sterilized and plated onto MS medium containing 50 mg/L hygromycin B. The plated seeds were vernalized at 4 °C for 2–4 days in the dark to synchronize germination and then transferred to a growth chamber at 22 °C (16/8 h photoperiod). 10-day-old *in vitro*-grown anti-hygromycin B seedlings were then transferred to soil mix. PCR and RT-PCR amplifications of the *ClCASPL* gene from candidate transgenic *Arabidopsis* plants were employed to confirm the successful transformation. WT and transgenic *Arabidopsis* T_3_ lines were used in this study. The SALK_034800C line was used for the screening of homozygous *AtCASPL4C1* knockout mutants of *Arabidopsis* using the three primers sets designed from the online service (http://signal.salk.edu/tdnaprimers.2.html). Primers used in this study for cDNA amplification of *ClCASPL* gene and screening of *AtCASPL4C1* knock-out mutant of *Arabidopsis* are listed in Supplementary Table 1.

### Casparian Strip Analysis

Propidium iodide (PI) staining was used to check Casparian strip formation in root as previously published procedures^9^. Roots from 5-day-old seedlings grown in MS medium were incubated in the dark for 10 min in 15 μM (10 μg/ml) PI (Invitrogen) and then were rinsed twice in water. The stained roots were observed using a fluorescence microscope (OLYMPUS BX51, Japan). Excitation and emission wavelengths are 488 nm and 500–550 nm.

### Chlorophyll Fluorescence Analysis

4-week-old seedlings of WT, *AtCASPL4C1* and *OX-ClCASPL* of plants were cold treated at 10 °C and Fv/Fm measurements were taken. Individual plants were dark-adapted for 20 min prior to measurement. Chlorophyll fluorescence was measured in dark-adapted plants at 0, 6, 12, 24, 48 and 72 h under 10 °C cold stresses using a chlorophyll fluorescence system (M-Series Imaging-PAM, Germany).

## Results

### Characterization of *CASPL*

**Figure 1:**
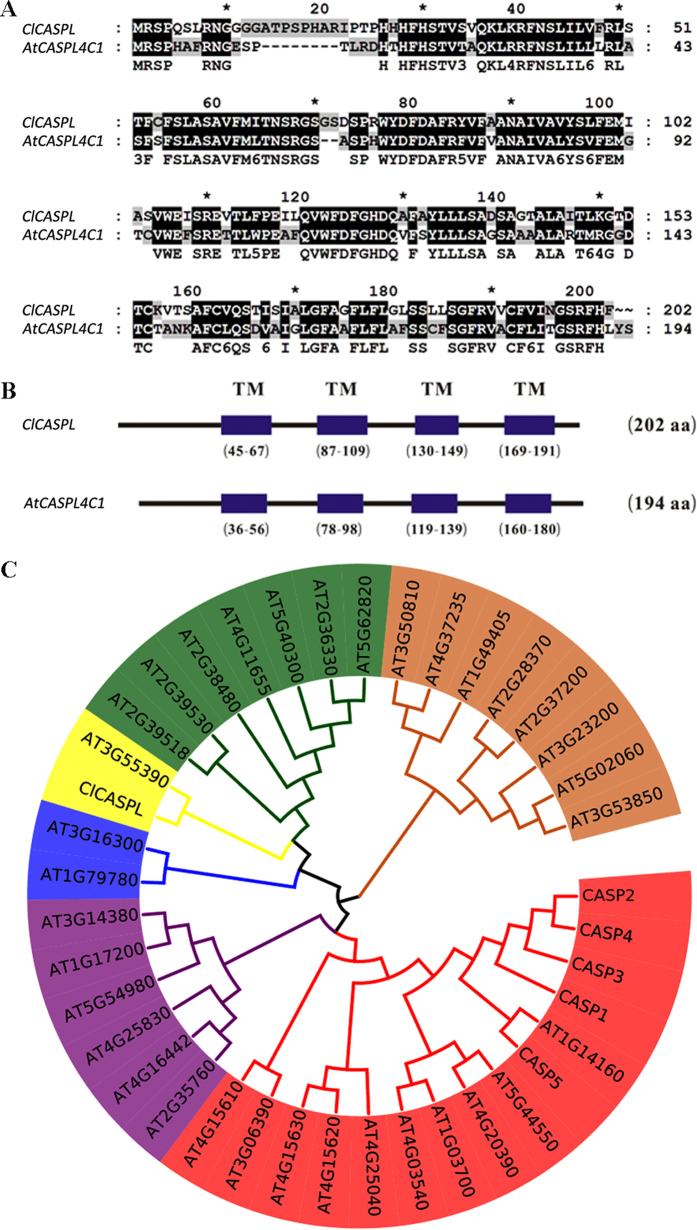
Characterization of the watermelon (Citrullus lanatus) ClCASPL and AtCASPL4C1 gene. (**A**) Protein sequence alignment of ClCASPL with AtCASPL4C1. (**B**) The four transmembrane (TM) regions of ClCASPL and AtCASPL4C1 predicated by the TMHMM program. (**C**) Phylogenetic tree of *ClCASPL* with *AtCASPL4C1* (*At3G55390*) and CASP family (UPF0497, 39 members, CASP1/2/3/4/5 have been identified as being involved with Casparian strip formation) from *Arabidopsis*.

A CASP-like integral membrane protein gene, *ClCASPL* (*Cla004012*), was isolated in watermelon (*Citrullus lanatus*) using RT-PCR and genome blasting (Supplementary Figure 1-A). Bioinformatics analysis showed that *ClCASPL* is orthologous to *At3g55390* from *Arabidopsis* (*AtCASPL4C1*, belonging to CASP family) (Fig. 1-A). Four transmembrane (TM) domains were predicted in ClCASPL (amino acids 45–67, 87–109, 130–149 and 169–191) and AtCASPL4C1 (At3g55390) proteins (amino acids 36–56, 78–98, 119–139 and 160–180 amino acid residues) by a variety of TM predication programs^5^ (Fig. 1-B). A total of 39 genes were identified that are defined as being part of the CASP family (UPF0497) in Arabidopsis, of which CASP1/2/3/4/5 has recently been identified to be associated with Casparian strip formation^9^. A phylogenetic tree for the CASP family in *Arabidopsis*^21^, defines 6 subfamilies using the Neighbor-Joining method and *ClCASPL* branches in the same subfamily as *At3g55390* (*AtCASPL4C1*) of *Arabidopsis* (Fig. 1-C).

### Subcellular Localization of ClCASPL Protein

**Figure 2:**
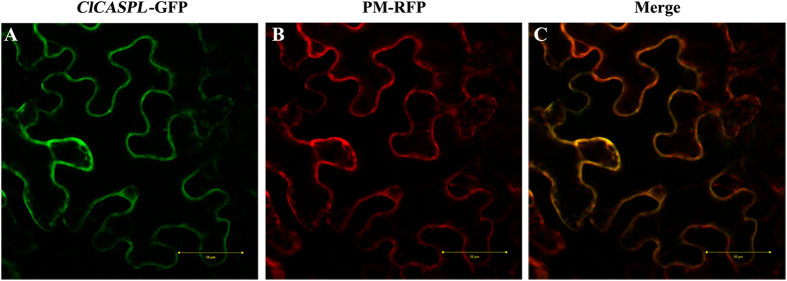
Subcellular localization of ClCASPL-GFP fusion protein in tobacco leaves. (**A**) Pattern of fluorescence for the ClCASPL-GFP fusion protein. (**B**) Pattern of fluorescence of the plasma membrane RFP marker. (**C**) Merged images of (**A**,**B**) showing that these overlap. Bar = 50 μm.

ClCASPL is predicted to be targeted to the plasma membrane, according to the TargetP and SignalP subcellular localization prediction programs^22^,^23^. The subcellular localization of ClCASPL was investigated by the generation of a ClCASPL-green fluorescent protein (GFP) fusion and transient expression in tobacco (*Nicotianaben thamiana*) leaves. Expression of the constructed *ClCASPL-GFP* gene revealed a fluorescence signal exclusively in the plasma membrane, co-localizing with the plasma membrane marker^16^ that was tagged with red fluorescent protein (RFP) (Fig. 2).

### Expression Analysis of *CASPL*

**Figure 3:**
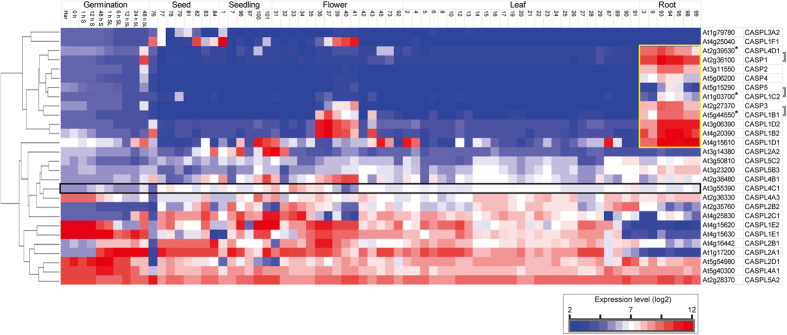
Transcript abundance profiles of 28 genes from the CASP protein family (UPF0497) that are represented on Affymetrix microarray chips. Transcript abundance is shown over germination and development. Expression levels (log_2_) are shown for the 28 genes (represented on microarrays) that encoding CASP or CASP-like genes. Hierarchical clustering shows strong co-expression of specific CASP like genes with known CASP genes (strongly co-expressed genes indicated by an asterisk*). The transcript abundance for *At3g55390* is shown in a black box, and genes that display a root enriched expression profile are shown in a yellow box. White boxes indicate no expression was detected.

**Figure 4:**
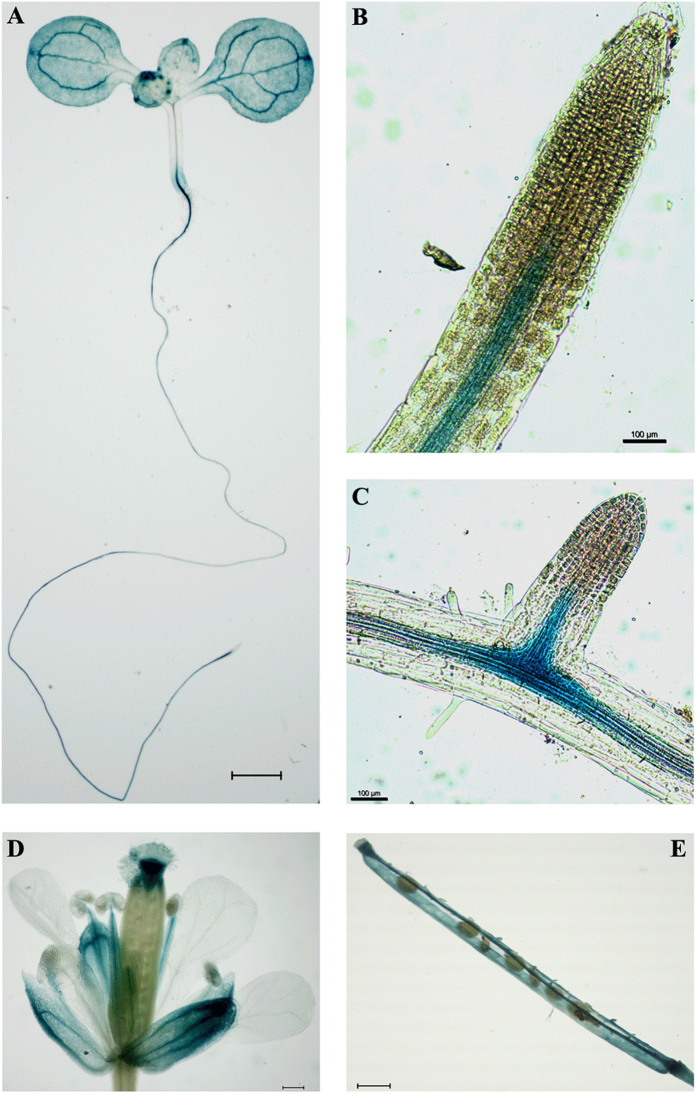
Histochemical analysis of CASPL gene expression in Arabidopsis. (**A**) Seedling and root of 1-week-old plants. (**B**) Root tip. (**C**) Lateral root. (**D**) Flower. (**E**) Silique. Bars = 1 mm in (**A,D,E**).

Transcriptomic analysis of public microarrays from the AtGenExpress developmental dataset^5^ and over seed germination^17^ indicated that *AtCASPL4C1* (*At3G55390*) is expressed in various tissues, but unlike some genes in this family it could not be defined as being predominantly expressed in root tissues (Fig. 3, black box). In contrast, a strong root expression profile is observed for several genes in this family, including CASP1/2/3/4/5 and other CASP-like genes (Fig. 3, yellow box). To determine spatial expression more precisely *AtCASPL4C1* expression patterns were determined using an *AtCASPL4C1*-promoter-GUS (for β-glucuronidase) reporter. Histochemical analysis expression in *planta*, with 12 independent lines all showed a very similar expression pattern, varied only slightly in the intensity of GUS staining. GUS-staining revealed that *AtCASPL4C1* expression is visible in roots and leaves (Fig. 4A). We can observe *AtCASPL4C1* expression in the vascular cylinder of roots, but no expression was detected in root tip (Fig. 4A,B). *AtCASPL4C1* expression was also detected in emerged lateral root (Fig. 4C). The filament, stigma and sepal of flowers also displayed intense staining (Fig. 4D), and signal could also be readily detected in siliques, but not in seeds (Fig. 4E). These results indicate that *AtCASPL4C1* is expressed in a wide range of variety of tissues and is not restricted to the vascular tissue of roots alone.

### *CASPL* Negatively Regulates Growth Dynamics

**Figure 5:**
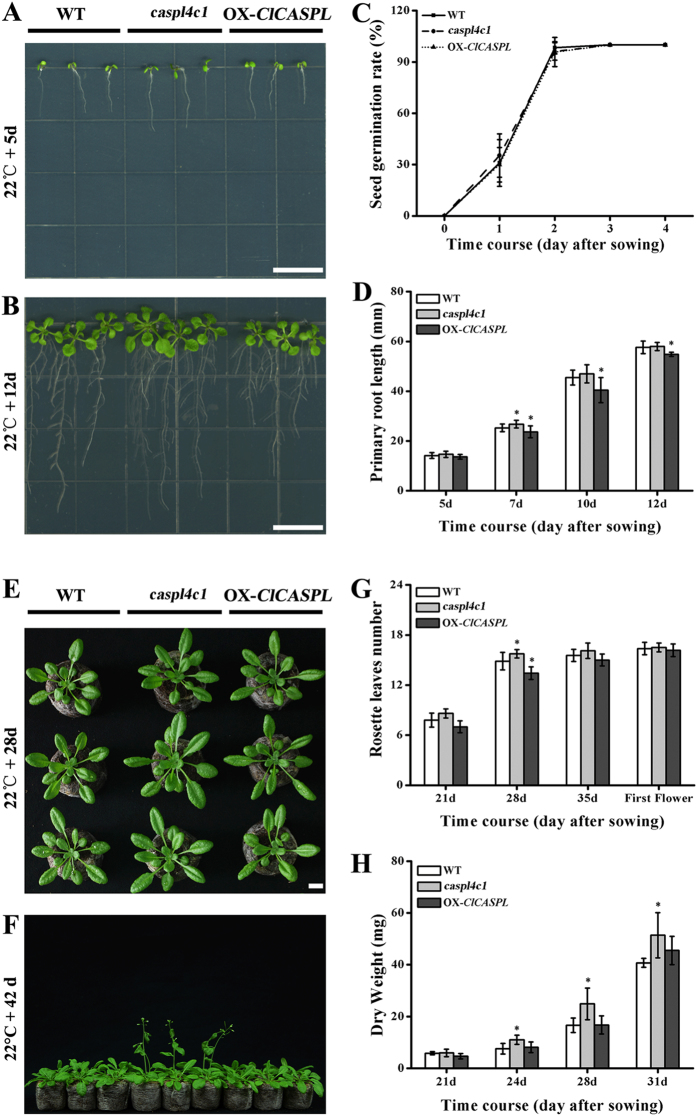
Phenotypes of wild type, AtCASPL4C1 and OX-ClCASPL under normal growth condition. (**A**) WT, *AtCASPL4C1* and OX-*ClCASPL* growth for 5 days in MS medium. (**B**) Wild type, *AtCASPL4C1* and OX-*ClCASPL* growth for 12 days. (**C**) Time course of germination for wild type, *AtCASPL4C1* and OX-*ClCASPL* in medium. (**D**) Time course of primary root length for WT, *AtCASPL4C1* and OX-*ClCASPL* in medium. (**E**) Wild type, *AtCASPL4C1* and OX-*ClCASPL* growth for 28 days in Jiffy seedling culture substrate. (**F**) Wild type, *AtCASPL4C1* and OX-*ClCASPL* growth for 42 days in Jiffy seedling culture substrate. (**G**) Time course of rosette leaves for wild type, *AtCASPL4C1* and OX-*ClCASPL* in Jiffy seedling culture substrate. (**H**) Time course of dry weight for WT, *AtCASPL4C1* and OX-*ClCASPL* in Jiffy seedling culture substrate. All growths are under growth condition of 22 °C and 16/8 h photoperiod in growth chamber. The star shows significance at 0.05 by Tukey test.

To investigate the function of *CASPL* in plants, *AtCASPL4C1* knockout mutants and transgenic plants overexpressing *ClCASPL* in *Arabidopsis* were generated (Supplementary Figure 1 and 2). Growth and development parameters were measured to quantify any changes in phenotype due to the absence or over-expression of *ClCASPL*. The germination rate among wild type (Col-0), *AtCASPL4C1* knockout and *OX-ClCASPL* plants did not significantly differ (Fig. 5A,C). The loss of *AtCASPL4C1*, however, did result in a small but significant increase in primary root length (Fig. 5D). In agreement with this, overexpressing *ClCASPL* in *Arabidopsis* significantly decreased primary root length (Fig. 5D). *AtCASPL4C1* plants displayed significantly faster growth in several parameters, bigger plants (Fig. 5B,E), more biomass (Fig. 5H) and earlier rosette leaves development and flowering (Fig. 5F,G) compared to wild type (Col-0) and *OX-ClCASPL* plants. Thus, the loss of *AtCASPL4C1* resulted in a positive growth affect, whereas over-expression gave the opposite effect. Thus, while only a single T-DNA insertion mutant is available for this gene in *Arabidopsis*, the complementary results of the knock-out and over-expressing plants strongly indicate that the phenotypes observed are due to the absence or increased abundance of *CASPL*.

### Casparian Strip Formation Analysis

In the CASP family (UPF0497) of *Arabidopsis*, *CASP1/2/3/4/5* (Casparian strip membrane domain), were identified to mediate Casparian strip formation in the endodermis^9^. The Casparian strip is made up of a lignin polymer without suberin in *Arabidopsis*^11^. Detection of Casparian strip formation by lignin staining in the root endodermis of *AtCASPL4C1*, wild type and *OX-ClCASPL* clearly indicated lignin staining in root endodermis of *AtCASPL4C1*, WT and *OX-ClCASPL* (Supplementary Figure 3A). *CASP1/2/3/4/5* transcript abundance was examined in root of wild type, *AtCASPL4C1* and *OX-ClCASPL*. The transcript abundance of *CASP1* was significantly altered, with increased transcript abundance in *AtCASPL4C1* knock-outs and reduced abundance observed in *OX-ClCASPL*. The transcript abundance of *CASP2*, *CASP3*, *CASP4* and *CASP5* were significantly increased transcript abundance in *AtCASPL4C1* knock-outs (Supplementary Figure 3B).

### *CASPL* Negatively Mediated Plant Tolerance to Cold Stress

The transcript abundance of CASP protein family was examined under various kinds of stresses in Arabidopsis by transcriptomic analysis. These abiotic stresses result in significant up-regulation/down-regulation of the greatest number of CASP and CASP-like genes (Supplementary Figure 4).

**Figure 6:**
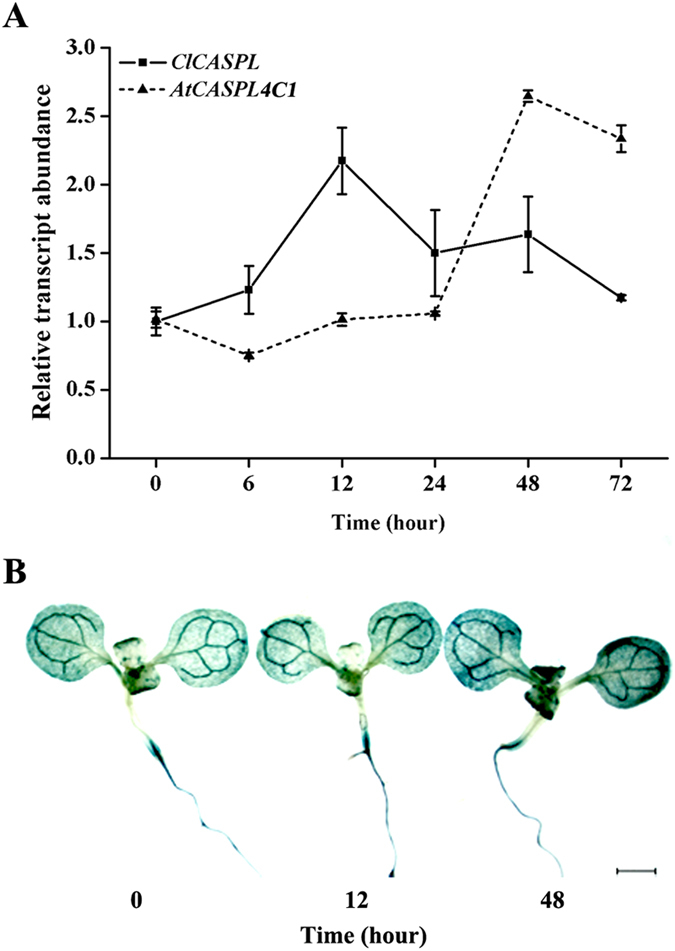
CASPL gene expression after cold stress. (**A**) Relative transcript abundance of *ClCASPL* in watermelon (real line) and *AtCASPL4C1* in *Arabidopsis* (dash line) over the course of 72 h of cold stress at 10 °C. (**B**) GUS staining of CASPL-promoter after cold stress (10 °C) for up to 48 h in Arabidopsis seedlings.

**Figure 7:**
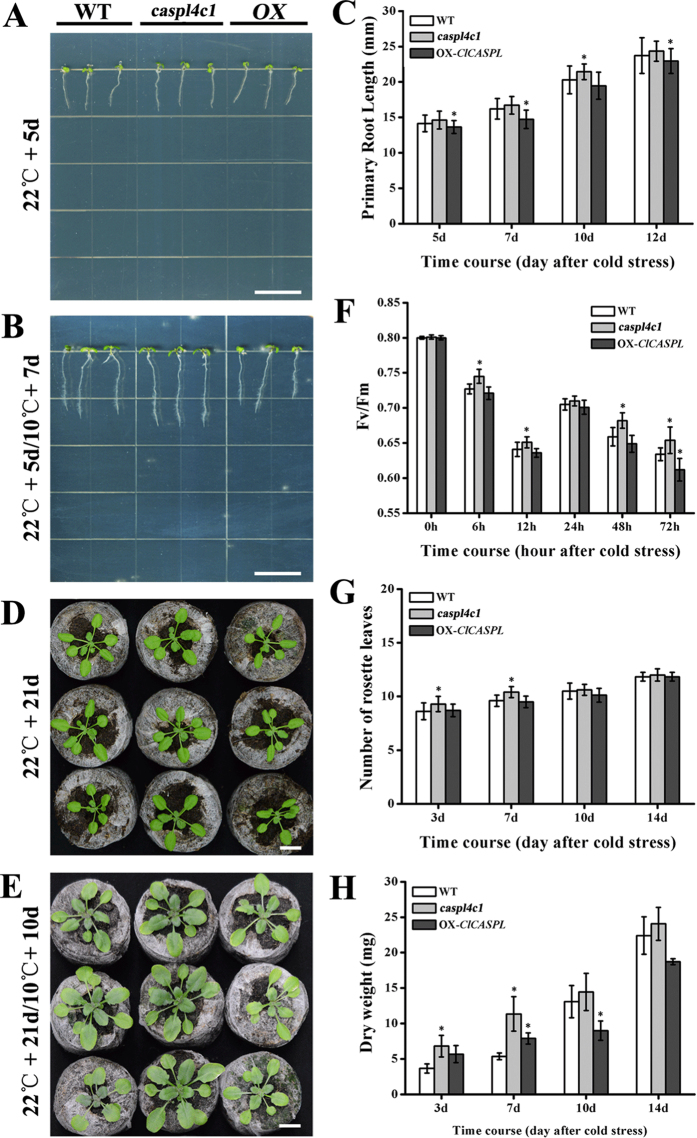
Phenotypes of wild type, AtCASPL4C1 and OX-ClCASPL under cold stress. (**A**) Wild type, *AtCASPL4C1* and OX-*ClCASPL* seedling growth for 5 days at 22 °C in MS medium. (**B**) 5-day-old seedlings of wild type, *AtCASPL4C1* and OX-*ClCASPL* grown for 7 days under cold stress at 10 °C in medium. (**C**) Time course of primary root length measurements for 5-day-old seedlings of wild type, *AtCASPL4C1* and OX-*ClCASPL* grown for 7 days at 10 °C in medium. (**D**) Wild type, *AtCASPL4C1* and OX-*ClCASPL* grown for 21 days at 22 °C in Jiffy seedling culture substrate. (**E**) 21-day-old seedlings of wild type, *AtCASPL4C1* and OX-*ClCASPL* grown for 10 days at 10 °C in Jiffy seedling culture substrate. (**F**) Time course of Fv/Fm fluorescence values after cold treatment at 10 °C in 21-day-old seedlings of WT, *AtCASPL4C1* and OX-*ClCASPL* plants. (**G**) Time course of rosette leaves for 21-day-old seedlings of WT, *AtCASPL4C1* and OX-*ClCASPL* after cold stress at 10 °C in Jiffy seedling culture substrate. (**H**) Time course of dry weight for 21-day-old seedlings of WT, *AtCASPL4C1* and OX-*ClCASPL* after cold stress at 10 °C in Jiffy seedling culture substrate. All growths are under growth condition of 16/8 h photoperiod in growth chamber. The star shows significance at 0.05 by Tukey test. OX represent OX-ClCASPL.

The transcript abundance of *ClCASPL* and *AtCASPL4C1* was examined under cold stress in watermelon and Arabidopsis. In watermelon, *ClCASPL* was induced and peaked in expression 12 h after cold treatment, while in Arabidopsis, *AtCASPL4C1* was induced and peaked in expression 48 h after cold treatment (Fig. 6A). Moreover, the *AtCASPL4C1* promoter-GUS activities after cold stress showed that expression of *AtCASPL4C1* was induced by cold stress in *Arabidopsis* (Fig. 6B). To study the role of *CASPL* in cold tolerance, different parameters were used to evaluate cold tolerance in wild type, *AtCASPL4C1* and *OX-ClCASPL* plants. Growth was monitored for 5-day-old plants exposed to cold at 10 °C for 7 days in medium. This examination showed that *AtCASPL4C1* grows better under cold stress, based on the significantly longer primary root length (Fig. 7A–C). Furthermore, 3-week-old *AtCASPL4C1* plants showed enhanced growth treated with cold stress, at 10 °C for 10 days (Fig. 7D,E), keeping relatively higher chlorophyll fluorescence parameters compared to WT (Fig. 7F), as well as, more rosette leaves and greater biomass (Fig. 7G,H). These results displayed greater cold tolerance than wild type and *OX-ClCASPL*.

## Discussion

The Casparian strip in the root endodermis is a barrier that restricts the free diffusion of molecules between the inner cell layers of the root. Recent studies revealed that this endodermal barrier is not only a regulator of water and nutrient uptake, but also probably acts as signal center for hormone-mediated control of growth^24^,^25^. It may be considered as a signal communication hub in response to the external environment, facilitating the activation of hormone signaling pathways and the propagation of calcium waves^24^,^25^. It also plays a central role in mediating cross talk between different cell layers during the development of new lateral organs, like lateral root emergence^26^. The molecular mechanisms involved in the deposition of the Casparian strip in endodermal cells are beginning to be uncovered^27^,^28^. The Casparian strip domain protein (CASP) anchors the plasma membrane to the cell wall and recruits the enzymes necessary for lignin biosynthesis^27^,^28^.

Evolutionary analysis indicated plant CASP family is associated with the MARVEL protein family described only in metazoans with similarity in specific transmembrane domains, in the overall tetraspan protein structure and putative Cys bridge in the second extracellular loop^12^. CASPs in this family are the earliest known proteins responsible for the CSD (Casparian strip membrane domain) formation^9^. The localizing at the CSD when expressed in the endodermis of CASPLs in this family prompts us to predict the role of yet-undiscovered CASPLs in mediating membrane subdomain formation and other plasma membrane domains in other cell types^12^. Here, we identified the membrane-localizing *AtCASPL4C1* and *ClCASPL* genes that encode proteins of the CASP family. Overall, the absence of AtCASPL4C1 results in significantly increased growth under normal conditions. Based on analysis of *CASP1/2/3/4/5* transcript abundance and lignin staining in roots, it is suggested that mutation in *AtCASPL4C1* (*At3G55390*) did not dramatically affect the Casparian strip formation in roots. It has previously been shown in *Atcasp1* or *Atcasp3* single mutants, that Casparian strip formation was unaltered compared to WT, however, *casp* double mutants (*casp1*/*casp3*) displayed disorganized Casparian strip^9^. Thus, due to redundancy it is not possible to conclude if there is a direct role in Casparian strip formation for AtCASPL4C1. However, loss of function of AtCASPL4C1 was observed to result in earlier vegetative and reproductive development and increased biomass (Fig. 5).

Plants respond to environmental changes by altering tissue differentiation and development, referred to as plant phenotypic plasticity. Here, we showed *CASPL* gene also regulates enhanced growth under cold stress conditions. While AtCASPL4C1 belongs to this family based on predicted protein homology, the expression pattern based on *in silico* analyses and *GUS* reporter gene expression patterns suggest that the role of AtCASPL4C1 may extend well beyond the Casparian strip in roots (Figs 3 and 4). The widespread expression pattern in vascular tissues also suggests that it plays a more fundamental role in plant growth and development, which is supported by the altered phenotype when it is inactivated or over-expressed.

In conclusion, we identified a CASP-like *ClCASPL* gene in *Citrullus lanatus* and its ortholog *AtCASPL4C1* in *Arabidopsis* are associated with growth and cold tolerance. These findings offered us clues to further explore the distinguished role of the Casparian strip protein family genes in growth, development and environmental communications.

## Additional Information

**How to cite this article**: Yang, J. *et al*. A Casparian strip domain-like gene, *CASPL*, negatively alters growth and cold tolerance. *Sci. Rep*. **5**, 14299; doi: 10.1038/srep14299 (2015).

## Supplementary Material

Supplementary Information
