# Supplementary material for: A Casparian strip domain-like gene, CASPL, negatively alters growth and cold tolerance
Source: Sci Rep. 2015 Sep 24;5:14299. doi: 10.1038/srep14299 (PMC4585827; doi:10.1038/srep14299)
Supplement: Supplementary Information [file srep14299-s1.pdf]

A Casparian strip domain-like gene, *CASPL*, negatively alters growth and cold tolerance

Jinghua Yang[1,2§], Changqing Ding[1,2§], Baochen Xu[1,2], Cuiting Chen[1,2], Reena Narsai[3], Jim Whelan[3], Zhongyuan Hu[1,2] and Mingfang Zhang[1,2]*

[1]Laboratory of Germplasm Innovation and Molecular Breeding, Institute of Vegetable Science, Zhejiang University, Hangzhou, 310058, P. R. China.

[2]Key laboratory of Horticultural Plant Growth, Development & Quality Improvement, Ministry of Agriculture, Hangzhou, 310058, P. R. China.

[3]Department of Animal, Plant and Soil Science, School of Life Science, Australian Research Council Centre of Excellence in Plant Energy Biology, LaTrobe University, Bundoora, Victoria 3086, Australian.

[§] These authors contributed equally to this work

*Corresponding author: mfzhang@zju.edu.cn

Supplementary Figures Legends

Supplementary Figure 1. Construction of *OX-ClCASPL*. **A)** Amplification of *ClCASPL* gene from *Citrullus lanatus*. **B)** Schematic construction of *OX-ClCASPL*. **C)** Enzyme digestion checking of construction. **D)** PCR checking of *OX-ClCASPL*. **E)** RT-PCR checking of *OX-ClCASPL*.

Supplementary Figure 2. Identification of *AtCASPL4C1* knockout-mutant in *Arabidopsis*. **A)** Homozygous *AtCASPL4C1* mutant. **B)** *AtCASPL4C1* expression in WT and *AtCASPL4C1* of *Arabidopsis*. **C)** T-DNA insertion position.

Supplementary Figure 3. Casparian strip staining and transcript abundance of AtCASP1/2/3/4/5 expressions in roots. **A)** Casparian strip staining of wild type, *Atcaspl4c1* and *OX-ClCASPL*. **B)** Relative transcript abundance of *AtCASP1/2/3/4/5* in wild type, *AtCASPL4C1* and *OX-ClCASPL* plants. The star shows significance at 0.05 by Tukey test.

Supplementary Figure 4. Top ten stresses resulting in significant up-regulation/down-regulation of the greatest number of CASP and CASP-like genes.

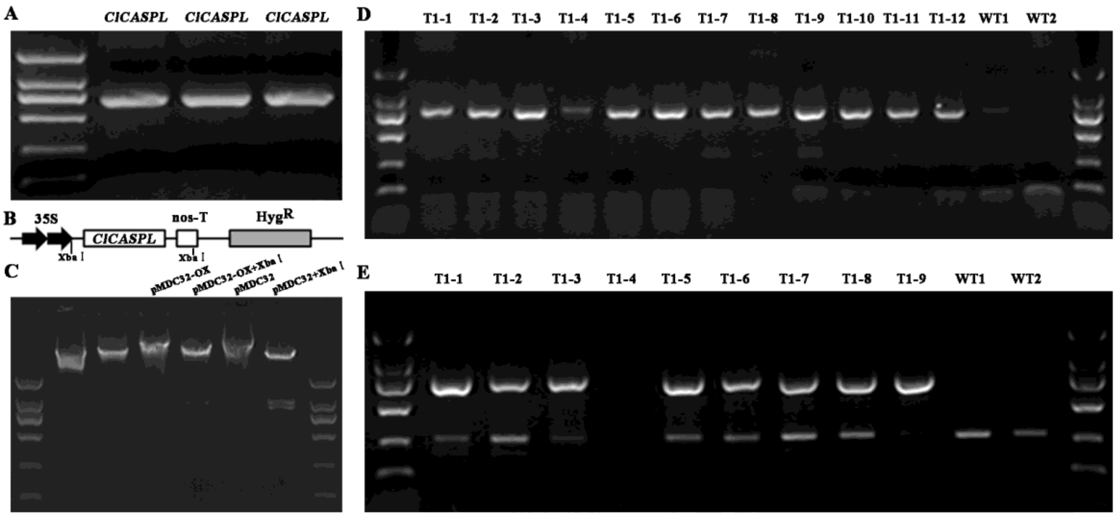
A
ClCASPL ClCASPL ClCASPL
B
35S
Xba I
ClCASPL
nos-T
Xba I
HygR
C
pMDC32-OX
pMDC32-OX+Xba I
pMDC32
pMDC32+Xba I
D
T1-1 T1-2 T1-3 T1-4 T1-5 T1-6 T1-7 T1-8 T1-9 T1-10 T1-11 T1-12 WT1 WT2
E
T1-1 T1-2 T1-3 T1-4 T1-5 T1-6 T1-7 T1-8 T1-9 WT1 WT2

Figure 1

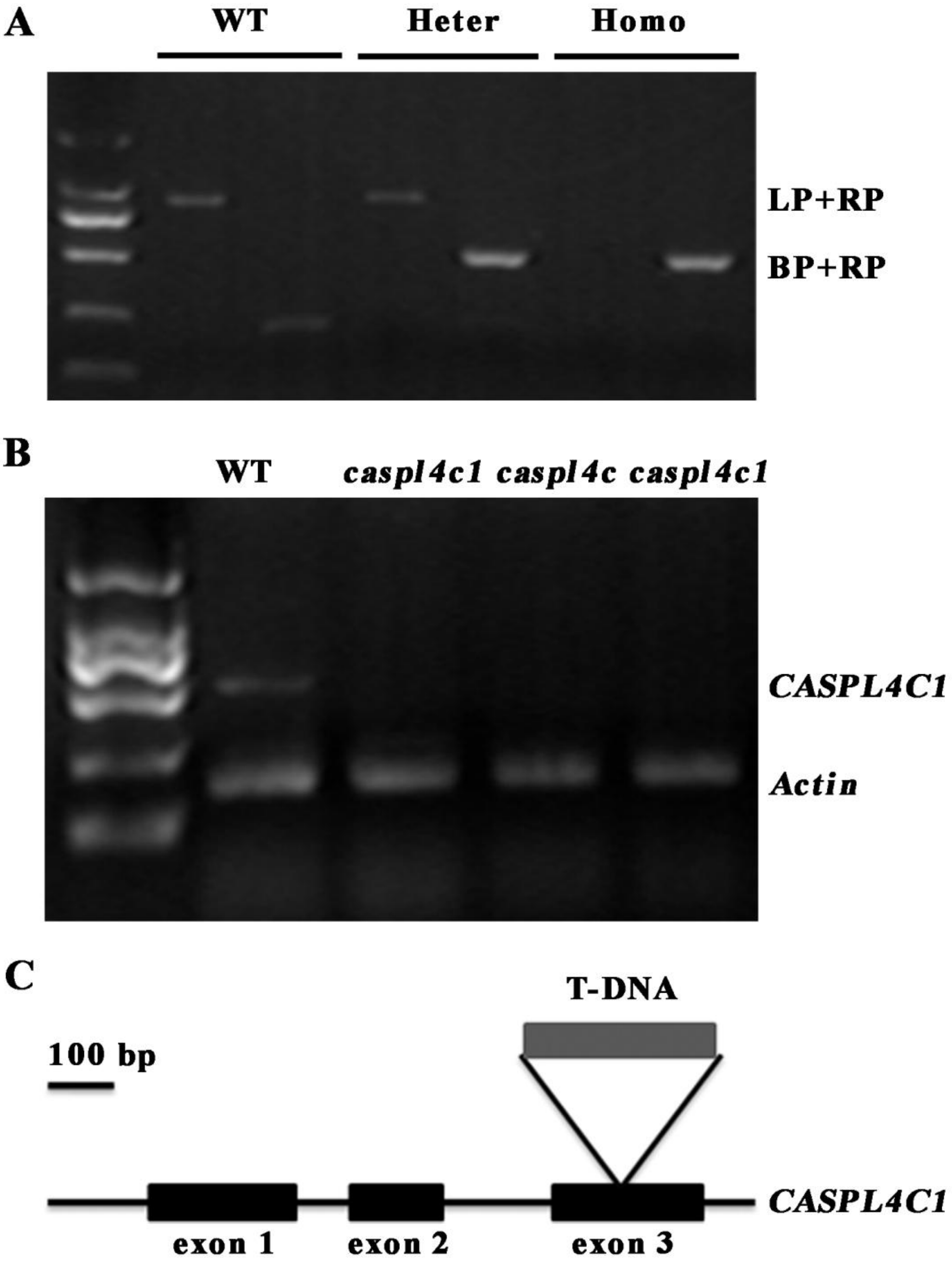
A
WT
Heter
Homo
LP+RP
BP+RP
B
WT
caspl4c1
caspl4c
caspl4c1
CASPL4C1
Actin
C
T-DNA
100 bp
CASPL4C1
exon 1
exon 2
exon 3

Figure 2

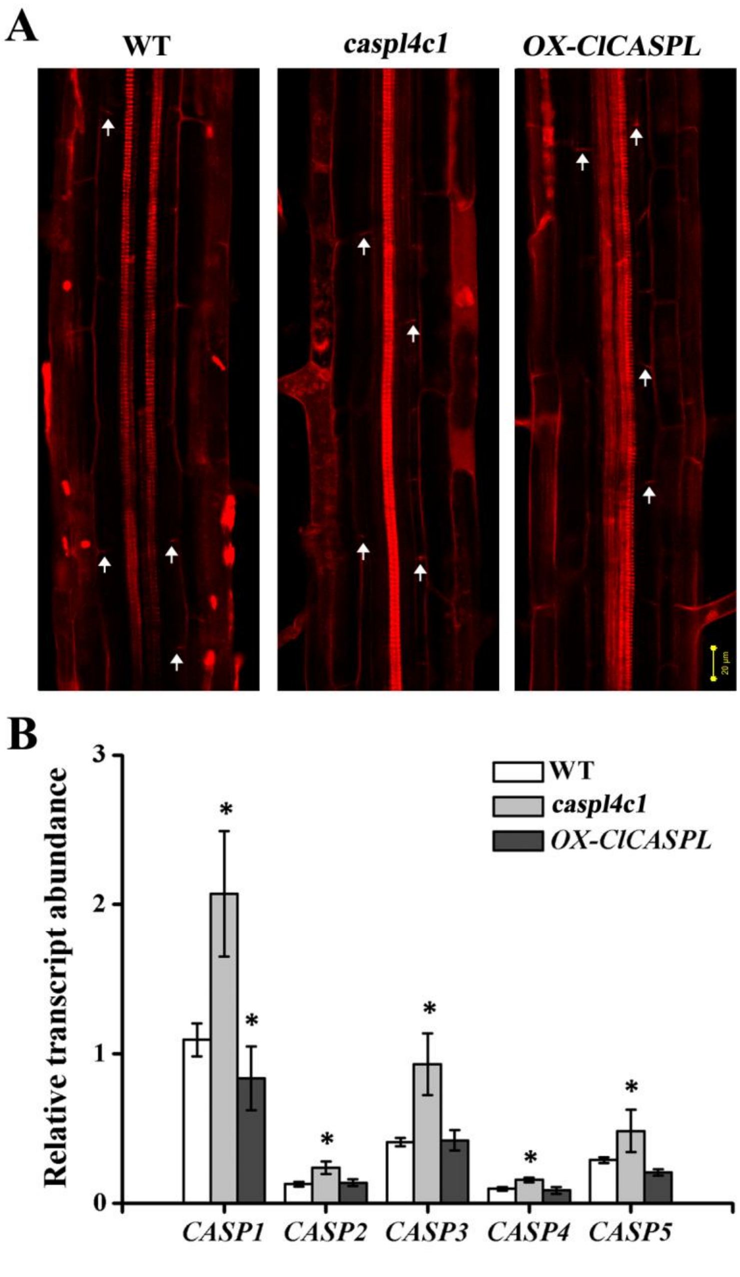
A
WT
caspl4c1
OX-ClCASPL
20 µm
B
Relative transcript abundance
0
1
2
3
WT
caspl4c1
OX-ClCASPL
CASP1
CASP2
CASP3
CASP4
CASP5

Figure 3

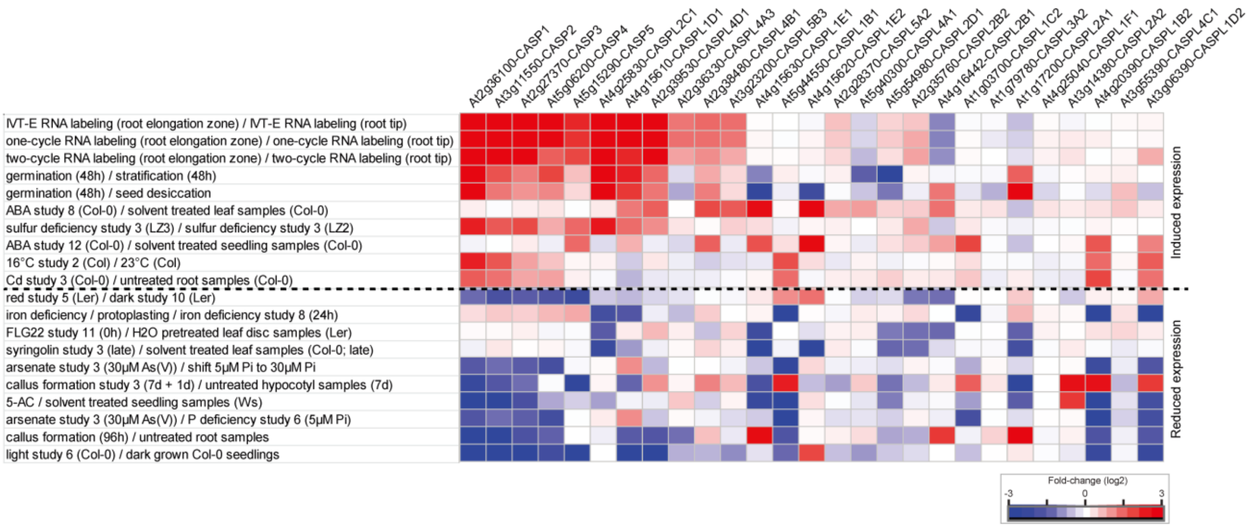
At2g36100-CASP1
At3g11550-CASP2
At2g27370-CASP3
At5g06200-CASP4
At5g15290-CASP5
At4g25830-CASPL2C1
At4g15610-CASPL1D1
At2g39530-CASPL4D1
At2g36330-CASPL4A3
At2g38480-CASPL4B1
At3g23200-CASPL5B3
At4g15630-CASPL1E1
At5g44550-CASPL1B1
At4g15620-CASPL1E2
At2g28370-CASPL5A2
At5g40300-CASPL4A1
At5g54980-CASPL2D1
At2g35760-CASPL2B2
At4g16442-CASPL2B1
At1g03700-CASPL1C2
At1g79780-CASPL3A2
At1g17200-CASPL2A1
At4g25040-CASPL1F1
At3g14380-CASPL2A2
At4g20390-CASPL1B2
At3g55390-CASPL4C1
At3g06390-CASPL1D2
IVT-E RNA labeling (root elongation zone) / IVT-E RNA labeling (root tip)
one-cycle RNA labeling (root elongation zone) / one-cycle RNA labeling (root tip)
two-cycle RNA labeling (root elongation zone) / two-cycle RNA labeling (root tip)
germination (48h) / stratification (48h)
germination (48h) / seed desiccation
ABA study 8 (Col-0) / solvent treated leaf samples (Col-0)
sulfur deficiency study 3 (LZ3) / sulfur deficiency study 3 (LZ2)
ABA study 12 (Col-0) / solvent treated seedling samples (Col-0)
16°C study 2 (Col) / 23°C (Col)
Cd study 3 (Col-0) / untreated root samples (Col-0)
red study 5 (Ler) / dark study 10 (Ler)
iron deficiency / protoplasting / iron deficiency study 8 (24h)
FLG22 study 11 (0h) / H2O pretreated leaf disc samples (Ler)
syringolin study 3 (late) / solvent treated leaf samples (Col-0; late)
arsenate study 3 (30µM As(V)) / shift 5µM Pi to 30µM Pi
callus formation study 3 (7d + 1d) / untreated hypocotyl samples (7d)
5-AC / solvent treated seedling samples (Ws)
arsenate study 3 (30µM As(V)) / P deficiency study 6 (5µM Pi)
callus formation (96h) / untreated root samples
light study 6 (Col-0) / dark grown Col-0 seedlings
Induced expression
Reduced expression
Fold-change (log2)
-3
0
3

Figure 4

Table 1 Primers used in this study

Gene	Forward primer	Reverse primer
ClCASPL-RT	CACCAATTTCTCCTCCATT	TCAAAACGACATGACGCACCA
Atcaspl4c1-Screen	GTTGCACCTTGCCTAAGTCTG (LP)	AGATTCGTGTTTGTGGCAAAC (RP)
	ATTTTGCCGATTTCGGAAC (LBa1.3)	
Transient-ClCASPL	CACCAATTTCTCCTCCATT	TCAAAACGACATGACGCACCA
AtCASPL4C1-RT	GTAACGGCGAATCTCCGACTT	AAACGAGAGCCAGTAATAAG
AtCASPL4C 1-qPCR	AGCTCAGAAACTCCGTCGAT	CTGAAAGCGTCGAAATCGTA
ClCASPL-qPCR	TGTTCAATCCACGATCTCCA	AACGAGAGCCGTTGATTACG
ClCASPL-GFP	AAAAAGCAGGCTCGCACCAATTTCTCCTCCATT	AGAAAGCTGGGTAATGAAAACGAGAGCCGTTG
AtCASPL4C 1-GUS	TAACGACTCCACTCCAGCAT	GCAGTCACGAGGCATCAA
CASP1-qPCR	CGAAGAAGAAGGGCTTTGTG	GCTTGGAACTGGAGGAACTG
CASP2-qPCR	CATGAAAGGCAAAGCTCCTC	CTGCAACAATGGCAGCTAAA
CASP3-qPCR	ACTCATCGCAGCAATCACAG	GTAGCCACCCACGATTGAGT
CASP4-qPCR	CATCAAAGGCAAAGCTCCTC	AATGGCAGCTAAACGGAGAA
CASP5-qPCR	TTAAGGCTGGAAGGTCGTTG	AGCGGTCTGATGAGCAGAAG
